# Living Systematic Reviews: An Emerging Opportunity to Narrow the Evidence-Practice Gap

**DOI:** 10.1371/journal.pmed.1001603

**Published:** 2014-02-18

**Authors:** Julian H. Elliott, Tari Turner, Ornella Clavisi, James Thomas, Julian P. T. Higgins, Chris Mavergames, Russell L. Gruen

**Affiliations:** 1 Department of Infectious Diseases, Alfred Hospital and Monash University, Melbourne, Australia; 2 School of Public Health and Preventive Medicine, Monash University, Melbourne, Australia; 3 World Vision Australia, Melbourne, Australia; 4 National Trauma Research Institute, Alfred Hospital, Melbourne, Australia; 5 EPPI-Centre, Institute of Education, University of London, London, England; 6 School of Social and Community Medicine, University of Bristol, Bristol, England; 7 Centre for Reviews and Dissemination, University of York, York, England; 8 Informatics and Knowledge Management Department, The Cochrane Collaboration, Freiburg, Germany; 9 Department of Surgery, Monash University, Melbourne, Australia

## Abstract

Julian Elliott and colleagues discuss how the current inability to keep systematic reviews up-to-date hampers the translation of knowledge into action. They propose living systematic reviews as a contribution to evidence synthesis to enhance the accuracy and utility of health evidence.

**Summary**

- The current difficulties in keeping systematic reviews up to date leads to considerable inaccuracy, hampering the translation of knowledge into action.
- Incremental advances in conventional review updating are unlikely to lead to substantial improvements in review currency. A new approach is needed.
- We propose living systematic review as a contribution to evidence synthesis that combines currency with rigour to enhance the accuracy and utility of health evidence.
- Living systematic reviews are high quality, up-to-date online summaries of health research, updated as new research becomes available, and enabled by improved production efficiency and adherence to the norms of scholarly communication.
- Together with innovations in primary research reporting and the creation and use of evidence in health systems, living systematic review contributes to an emerging evidence ecosystem.

## The Bridge from Evidence to Practice

Health research promises societal benefit by making better health possible. However, there has always been a gap between research findings (what is known) and health care practice (what is done), described as the “evidence-practice” or “know-do” gap [1]. The reasons for this gap are complex [2], but it is clear that synthesising the complex, incomplete, and at times conflicting findings of biomedical research into forms that can readily inform health decision making is an essential component of the bridge from “knowing” to “doing.”

**Figure 1:**
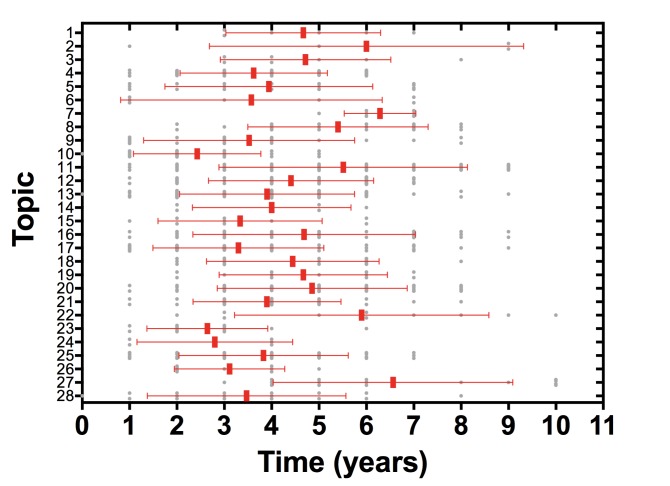
Time from primary study publication to incorporation in systematic review. Analysis of 792 study reports incorporated into 73 systematic reviews across 28 high priority topics in the field of neurotrauma. Study reports were included in the analysis if they were incorporated into a systematic review relevant to one of the high priority topics and published in the period 2001–2009. Systematic reviews were included in the analysis if they were relevant to one of the high priority topics and published in the period 2001–2012. Bars represent medians and interquartile range.

Systematic reviews (SRs) and meta-analyses have provided incalculable benefit for human health by contributing to the bridge from knowing to doing, but this benefit is limited by characteristics of the current SR enterprise [3]. The methods of SR and meta-analysis are well developed [3]–[5], but less progress has been achieved with the other essential component of accuracy—currency. The time from the date of the last search to SR publication is commonly over a year [6], and in an analysis of the time taken for primary study results to be incorporated into an SR, the median time from primary study publication to SR publication ranged from 2.5 to 6.5 years (Figure 1) [7]. Once published, only a minority of reviews are updated within 2 years of publication [8], and this inability to maintain currency leads to significant inaccuracy. By 2 years post-publication 23% of SRs that have not been updated will have failed to incorporate new evidence that would substantively change conclusions about the effectiveness or harms of therapies [9].

Current approaches to updating SRs focus on detecting SRs most in need of updating [10],[11]. While these methods contribute to the currency of SRs, they cannot adequately reduce the inaccuracy caused by out-of-date SRs. It is often difficult to assemble an authorship team to complete prioritised updates; publication of updates takes many months, during which time the SR remains out of date and therefore potentially inaccurate; and many SRs are not designated as high priority and therefore remain out of date and inaccurate. Despite the availability of these methods and the concerted efforts of many individuals, much of the global corpus of SRs remains out of date.

Incremental advances in traditional SR updating is unlikely to lead to substantial and sustained improvements in the currency of SRs in the context of exponential growth in primary research and SRs [12]. In order to address this considerable source of SR inaccuracy, and produce evidence summaries that are both methodologically rigorous and up to date, a new approach is needed. This innovation has previously been unachievable because rigorous reviews are demanding of time and resources, and “up to date” required rapid processes, which limited the feasibility of rigorous methods. New technologies create the opportunity to resolve this trade-off and enable answers to health questions that are both methodologically rigorous and up to date. We have named this new approach to the updating of SR “living systematic review.” Our aim in proposing this approach is to address the challenges facing contemporary evidence synthesis, while retaining the strengths of SR that have been a critical underpinning of knowledge for health over the last quarter century. This approach to SR adds to rather than replaces existing methods and is particularly relevant for reviews of controlled trials in fast moving topic areas.

## Living Systematic Reviews

Living systematic reviews are high quality, up-to-date online summaries of health research that are updated as new research becomes available, consistent with the vision of the pioneering Oxford Database of Perinatal Trials to “include a library of trial overviews, which will be updated when new data become available” [13]. There are four fundamental differences between conventional SR and living systematic review: publication format, work processes, author team management, and statistical methods.

The essential difference between living systematic review and conventional SR is the publication format. Instead of a conventional static SR report or update, living systematic reviews are dynamic, persistent, online-only evidence summaries, which are updated rapidly and frequently. The corollary of a living publication is three changes to SR production. First, work processes must be adapted. A literature search strategy is maintained and outputs fed continuously into an SR workflow, including continuous updating of identified studies, assessment of study quality, data extraction, meta-analysis, and SR report. Instead of the intense, sporadic effort of conventional SRs and SR updates, living systematic review requires moderate, ongoing contribution. Second, author team management must be responsive to a continuous workflow, coordinating effort over long periods of time and allowing evolution in the author team, while maintaining institutional memory.

Third, updating meta-analyses entails a reanalysis of data and, as with repeated analysis of accumulating primary trial data, an inflated rate of false-positive findings is likely if statistical tests are repeated naively [14]. Also, effect size estimates can be unstable, especially in the early stages of evidence generation [15]. These issues are relevant to all updates to meta-analyses, but are particularly important for living systematic reviews given the potential frequency of updates. Statistical monitoring of meta-analyses using formal sequential methods can control the risk of spurious findings, while achieving pre-specified power to identify a pre-specified clinically relevant magnitude of effect [16],[17]. Sequential methods are controversial in meta-analysis since they are based on testing rather than estimation, and an estimate that is widely disseminated on the basis of a significance test result may be biased. As an alternative, a Bayesian approach provides a natural framework for monitoring accumulating evidence in which prior distributions can be used to reduce the probability of falsely concluding superiority of an intervention in the early stages of the review [18], and to stabilize the meta-analysis by exploiting external information about the likely degree of statistical heterogeneity across the studies [19]. Furthermore, a Bayesian framework feeds naturally into decision making.

## Production of Living Systematic Reviews

In addition to the three essential changes to review production described above, a key enabler of living systematic review is SR production efficiency. Improvements in efficiency can have profound effects on the application of health technologies. For example, a 4-log decrease in the cost of sequencing a human genome, from US$100 million to US$10,000 over 10 years [20], has catalysed a genomics revolution with profound benefits for health. In contrast, rising methodological expectations have led to an increase in the complexity and cost of SR and production timelines often in excess of 1–2 years [21]. We describe below several recent developments that have the potential to improve dramatically the efficiency of conventional SR and enable the widespread production of living systematic reviews.

## 

### Workflow and Collaboration Tools

Despite the availability of some specific tools, the efforts of most SR authors are fragmented across generic word processing, spreadsheet, email, reference management, and statistical analysis tools [22],[23]. This fragmentation hampers the production and updating of SRs, undermines the experience and engagement of SR authors, and limits the availability of process data. Growing innovation in tools and platforms [22] will enable more efficient SR production, but the right incentives and partnerships need to be in place for these innovations to translate into broadly available applications [24].

### Semi-automation

Text-mining technologies are currently being developed to improve the efficiency of SR production [25]. While experimental work encompasses many stages of the review process the most refined techniques are currently concerned with study identification [26]. Here, machine learning technologies have the potential to reduce the manual “screening” of titles and abstracts by up to 50% in new reviews [27] and more than 90% in review updates [28], greatly enhancing the efficiency of review production. Other initiatives are developing semi-automation technologies to assist with the development of search strategies, assessment of study quality, extraction of data from documents, and production of SR protocols and reports.

### Data Repositories and Linked Data

Important health care questions are often the subject of evidence syntheses by multiple independent groups isolated from each other in redundant effort. The value embedded in the process and output of these parallel activities is only partially captured in discrete, static, and unstructured document-based outputs. Efforts to encourage registration of SRs can help minimise unnecessary duplication [29]. Initiatives that enable SR process and output data to be prospectively stored and reused by others are important developments that will reduce redundant effort [30],[31]. When these data are stored in structured formats using shared ontologies [32],[33], following W3C formats for linked data (RDF, OWL), unnecessary duplication can be avoided, but opportunities will also arise to draw from and contribute to the rapidly expanding world of linked open data.

### Participation and the Crowd

Despite the fact that SRs are important, resource intensive, and time critical, most SRs are conducted by small academic teams, working part-time over many months. Larger authorship groups increase the efficiency of SR production [34] and the expertise available to each review group [3], but remain underutilised, particularly for high priority questions in which both the demand for evidence and engagement are high. In clinical and laboratory research, high priority questions are often addressed by collaborations of dozens or hundreds of researchers working together, but similar undertakings do not currently exist in evidence synthesis. Increasing the involvement of end users in SR production improves the outputs of SR [35]–[37], and “citizen science” approaches in which citation screening is crowd-sourced from a network of non-expert contributors have also been tested [38]. Efforts to identify smaller units of scholarly effort for dissemination and attribution [39]–[41] may be applicable to SR and encourage broader participation. These approaches to participation need to be evaluated in comparison to conventional approaches and to manage the risk of bias associated with contributors' potential competing interests.

## Publication of Living Systematic Reviews

The shift to a persistent, dynamic online-only publication format will be enabled by at least two other developments. First is efficient peer and editorial review of a living document. When an ongoing search strategy identifies no new studies for inclusion, the review can be updated with the date of last search without further review. When new studies or data are identified for inclusion, but these make negligible difference to summary estimates and have no effect on review conclusions, these data can be incorporated into the review with a modest form of review (e.g., editorial only). If new studies or data are identified that result in significant changes to summary estimates or the review's conclusions, these should undergo rapid, but nonetheless robust, peer and editorial review. In the latter situations post-publication peer review can contribute to the accuracy of published reviews.

Second, living systematic reviews should be compatible with the norms of scholarly communication. Attribution of contribution to the living publication can follow existing norms, such as ICMJE criteria. Once authors' contributions no longer fulfil criteria for authorship, they can be removed from the author list and acknowledged as former contributors. Citation can also follow existing practices, including version number/date and date accessed. Finally, current practice can also inform listing in bibliographic databases with minor updates appended to an existing entry and major updates listed as a new publication.

## Living Systematic Review and a New Evidence Ecosystem

Living systematic review contributes to the translation of knowledge into practice, primarily because of the contribution currency makes to accuracy and utility, but this approach to evidence synthesis also supports, and is enhanced by, associated upstream and downstream innovations.

In the growing deluge of research the noble science of systematic review resembles archeology: academic teams searching for buried artifacts and working tirelessly to reveal their true meaning. The growth in primary research and availability of diverse research outputs—protocols, trial registration, clinical study reports, and individual patient data—will continue to challenge current SR models. New methods are needed to identify datasets relevant to specific health questions and enable synthesis and insight. For example, annotation of research outputs with richer meta-data will increase the efficiency of review-specific search and screening. More importantly, publication and verification of research outputs in structured forms (e.g., using semantic technologies) will transform review-specific quality assessment and data extraction [42]. Living systematic review, together with these upstream innovations, will ensure that the potential rich insights from large datasets such as clinical study reports, individual patient data, and health system “big data” are made available for health decision making in a rigorous, efficient, and timely manner.

**Figure 2:**
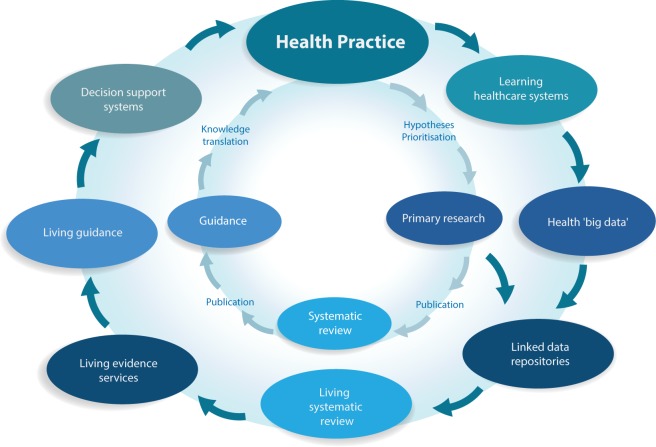
Current and emerging health knowledge ecosystems. The current health knowledge ecosystem (inner circle) is characterized by inefficiencies that hamper the flow of knowledge from health practice through primary research, systematic review and guidelines, and finally back to impacts on health practice. The new health knowledge ecosystem that is emerging (outer circle) is characterized by a continuous flow of knowledge between efficient, living components, including the growing importance of learning health care systems, which together with traditional primary research will populate common data repositories. Living evidence services derived from these repositories, supporting living guidance and decision support systems will close a “living” health knowledge loop.

Living systematic review also enhances the efficiency and opportunities for knowledge translation. First, living systematic review enables “living knowledge translation,” including living guidelines, standards, policies, and decision support systems. Second, the value inside the “container” of an SR can be unlocked when the associated data are made available, particularly as open access to linked data formats. The availability of these data opens up opportunities for integration with guideline development platforms and clinical decision support systems [43] to create a new evidence “ecosystem” (Figure 2).

## Conclusion

An essential link between health research and societal benefit is the transformation of millions of published research studies into accurate and usable summaries for health decision making. Many individuals and organisations are committed to this task and their efforts have improved the health of our societies, but increasing demand for accurate evidence—methodologically rigorous and up to date—is not being met. We propose living systematic review as a contribution to the methods of evidence synthesis that addresses these challenges by combining currency with rigour to enhance the accuracy and utility of health evidence. Living systematic review involves modifications to review production and publication, enabled by improved production efficiency and adherence to the norms of scholarly communication. The approach is widely applicable and although challenges remain (Table 1), feasible responses to these challenges exist. Together with emerging innovations in the reporting of primary research and in the creation and use of evidence in health systems, living systematic review contributes to a new evidence ecosystem in which health knowledge and practice are efficiently and rigorously entwined.

**Table 1:** Living systematic review: modifications to conventional systematic review and key challenges.

Category	Item	Description	Key Challenges
**Essential modifications to conventional processes**			
Production	Work processes	Search strategy maintained and fed continuously into SR workflow	Shift to continuous work processes
	Author team management	Coordinated and continuous effort	Coordination of ongoing review outputs and author team turnover
	Statistical methods	Updating of meta-analysis	Building consensus regarding appropriate statistical methods
Publication	Publication format	Persistent, dynamic online-only publication	Updatable online publication formats with authorship and version control
**Enablers**			
Production	Workflow and collaboration tools	Tools and platforms for SR authoring	Incentives and partnerships for innovation and translation into widely available applications
	Semi-automation	Machine-assisted SR production processes	Demonstration of performance in large scale real world implementation; extension of applications beyond citation screening
	Data repositories and linked data	Repositories of structured SR data	Development of common data formats, controlled vocabularies and SR ontologies
	Participation and the crowd	Larger, diverse author groups; citizen and crowd participation; nanopublication	Demonstration of performance in large scale real world implementation; refining incentives, training, and quality assurance
Publication	Peer and editorial review	Adaptations to conventional peer and editorial review	Validation and acceptance by academic community
	Norms of scholarly communication	Attribution, citation, and listing in bibliographic databases	Ensuring conventional academic incentives are maintained
**Associated developments**			
Production	Research output annotation	Annotation of research outputs with rich, structured meta-data	Development of meta-data, processes, and incentives
	Structured research datasets	Publication and verification of structured research data	Standards, models, and incentives for data sharing
Publication	Living knowledge translation	Living guidelines, standards, policies, and decision support systems	Development, demonstration of feasibility, and evaluation of benefits and risks
	Integration with guideline development and decision support systems	Structured data moving “nimbly” from SR to guideline to decision support systems	Development, validation, and evaluation of integrated evidence systems
